# The Surprise Question: Not Ready for Prime Time

**DOI:** 10.1089/pmr.2024.0071

**Published:** 2024-10-09

**Authors:** Mellar P. Davis

**Affiliations:** Geisinger Medical Center, Danville, Pennsylvania, USA.

**To the editor**

I read the article by Dr. Coulon and colleagues with interest (Palliative Medicine Reports Volume 5.1.2024). They explored the use of the Surprise Question in geriatric patients admitted through the emergency department and compared surprise question assessments and mortality outcomes between nurses and physicians. The findings are very much in line with a review we previously published.

I am particularly intrigued by these findings and am eager to discuss them further.^1^ It is also consistent with the data from our medical center. Their positive likelihood ratio was approximately 2.4 to 2.3. This translates to an additional 15% mortality prediction. A positive likelihood ratio of 2–5 has weak evidence to rule in mortality. A negative likelihood ratio of 0.1–0.2 has moderate evidence against mortality, and a ratio of 0.2–0.5 is weak evidence.^2^ The Surprise Question, when used alone, is a weak prognostic tool. Two recent systematic reviews used specificity, sensitivity, predictive values and not likelihood ratios.^3,4^ Predictive values are highly dependent on the prevalence of mortality in the population and, hence, are not an accurate means of determining the diagnostic capability of a tool. The clinical relevance to the C-statistic is abstract, unlike the likelihood ratios. The original intent of the surprise question was a referral to palliative care if the question was answered “positive,” and yet most studies have used it as a prognostic tool. There is no information on the palliative and economic outcomes of using the Surprise Question as a referral. The Surprise Question may add to other prognostic factors.^5^ A retrospective study of 535 patients with heart failure found that combining the Surprise Question and the number of heart failure admissions improved the specificity compared with hospitalizations and the Surprise Question alone.^6^ Future studies should explore the use of the Surprise Question with other prognostic factors. Clinical care should not be based on the Surprise Question until patient, caregiver, and health care outcome benefits are realized when using the Surprise Question.

## References

[1] Davis MP, Vanenkevort E. The Surprise quesion. BMJ Support Palliat Care 2022;12(4):403–406. 10.1136/spcare-2022-003853 36038254

[2] Davis MP, Soni K, Strobel S. Likelihood ratios: An important concept for palliative physicians to understand. Am J Hosp Palliat Care 2023;40(8):894–899. 36202637 10.1177/10499091221132454

[3] van Lummel EV, Ietswaard L, Zuithoff NP, et al. The utility of the surprise question: A useful tool for identifying patients nearing the last phase of life? A systematic review and meta-analysis. Palliat Med 2022;36(7):1023–1046. 35769037 10.1177/02692163221099116 PMC10941345

[4] Gupta A, Burgess R, Drozd M, et al. The surprise question and clinician-predicted prognosis: Systematic review and meta-analysis. BMJ Support Palliat Care 2024. 10.1136/spcare-2024-004879 PMC11874281 38925876

[5] Davis MP. Letter to the Editor: The surprise question in palliative care: Does it have a role in predicting mortality? J Palliat Med 2024;27(3):292–293. 38427904 10.1089/jpm.2023.0677

[6] Blum M, Gelfman LP, McKendrick K, et al. Enhancing palliative care for patients with advanced heart failure through simple prognostication tools: A comparison of the surprise question, the number of previous heart failure hospitalizations, and the seattle heart failure model for predicting 1-year survival. Front Cardiovasc Med 2022;9:836237. 35479267 10.3389/fcvm.2022.836237 PMC9035562

